# Degrading habitats and the effect of topographic complexity on risk assessment

**DOI:** 10.1002/ece3.793

**Published:** 2013-09-30

**Authors:** Mark I McCormick, Oona M Lönnstedt

**Affiliations:** ARC Centre of Excellence for Coral Reef Studies, and School of Marine and Tropical Biology, James Cook UniversityTownsville, Qld, 4811, Australia

**Keywords:** Chemical alarm cue, coral reef fish, disturbance, olfactory cues, predator recognition, sensory compensation, visual cues

## Abstract

Topographic complexity is a key component of habitats that influences communities by modulating the interactions among individuals that drive population processes such as recruitment, competition, and predation. A broad range of disturbance agents affect biological communities indirectly through their modifications to habitat complexity. Individuals that best judge the threat of predation within their environment and can trade-off vigilance against behaviors that promote growth will be rewarded with the highest fitness. This study experimentally examined whether topographic habitat complexity affected the way a damselfish assessed predation risk using olfactory, visual, or combined cues. Fish had higher feeding rates in the low complexity environment. In a low complexity environment, damage-released olfactory cues and visual cues of predators complemented each other in the prey's assessment of risk. However, where complexity was high and visual cues obscured, prey had lower feeding rates and relied more heavily on olfactory cues for risk assessment. Overall, fish appear to be more conservative in the high complexity treatment. Low complexity promoted extremes of behavior, with higher foraging activity but a greater response to predation threats compared with the high complexity treatment. The degree of flexibility that individuals and species have in their ability to adjust the balance of senses used in risk assessment will determine the extent to which organisms will tolerate modifications to their habitat through disturbance.

## Introduction

Most natural habitats undergo frequent disturbance from biological and environmental agents (e.g., Mumby et al. 2011; Brodie et al. 2012), and individuals must continuously adapt and react to their changing environment or die. As the environment changes, the ways the prey assess the risk of predation are predicted to change as the lucidity of sensory cues will be strongly tied to prevailing habitat features. For instance, storm damage may modify a forest canopy and understory, thereby affecting the distances at which predators and their prey can visually detect one another (Metcalfe 1984). A commonly held misconception is that complex habitats are always safer for prey species because of the abundance of hiding places. By mediating the detection of predators by prey, topographic complexity affects a range of trait-mediated predator-induced effects, such as elevated stress levels and reduced body condition (Schoener et al. 2002; Clinchy et al. 2013). Organisms that are successful in adapting to the new and unfamiliar habitat must modify the balance of cues that they will use to assess risk (i.e., “sensory compensation”; Hartman and Abrahams 2000). Literature suggests that terrestrial habitats with low topographic complexity may have high range of risk (Laundré et al. 2001; Creel and Christianson 2007); safe places are more secure, and dangerous places are riskier in open habitats compared with topographically complex habitats. While there is a well-documented link between reductions in habitat complexity and reduced species diversity (Hewitt et al. 2005; Leal et al. 2012), the extent to which this relationship is driven by changes in the way the prey assess predation risk is unknown.

Coral reefs are the poster child for a topographic complex ecosystem with high species diversity. However, it is also an ecosystem that experiences high levels of disturbances from such vectors as crown of thorns starfish, coral bleaching, coral disease, and cyclonic events (e.g., Moran 1986; Willis et al. 2004; Thompson and Dolman 2010). Recently, this ecosystem has come under intense scrutiny because of its vulnerability to the predictions of elevated temperature, increased storm damage, and modified ocean chemistry associated with CO_2_ induced climate change (Wilson et al. 2006; Hughes et al. 2010). Bleaching of reef-building corals is predicted to increase (Anthony et al. 2011) and has already been linked to dramatic reductions in the standing stock of fishes and their diversity (Jones et al. 2004; Pratchett et al. 2009; Wilson et al. 2009). The proximate mechanism for these reductions may be lowered topographic complexity (e.g., Pratchett et al. 2008; Alvarez-Filip et al. 2009; Figure S1), which alters key population processes such as recruitment and predation (Jones et al. 2004; Munday et al. 2008; Pratchett et al. 2008). Currently, it is unknown how topographic complexity influences predator–prey interactions through modifications in risk perception for this species-rich ecosystem.

The main senses used for risk assessment in the aquatic environment are visual and chemical cues (Lönnstedt et al. 2012), and topographic complexity can be expected to affect these differentially. The “sensory compensation hypothesis” predicts that when visual cues are limited, the response to chemical information should be accentuated (Lima and Steury 2005; Ferrari et al. 2008). Similarly, visual cues are expected to be more important for risk assessment in environments where visibility is relatively high, such as in areas of low structural complexity (Chivers et al. 2001). We know very little about how reductions in habitat topography will affect the balance of information obtained from these two crucial senses. The aim of this study was therefore to experimentally examine how topographic complexity affected the balance of visual and chemical cues used to assess predation risk by a common coral reef damselfish, *Pomacentrus amboinensis*. We predicted that the damselfish would compensate for poor visibility in topographically complex habitats by relying more on chemical information to assess risk. We also expected that sensory compensation would allow prey fish to respond stronger to visual cues of a predator in areas of low complexity.

## Materials and Methods

### Study species and sampling

**Figure 1:**
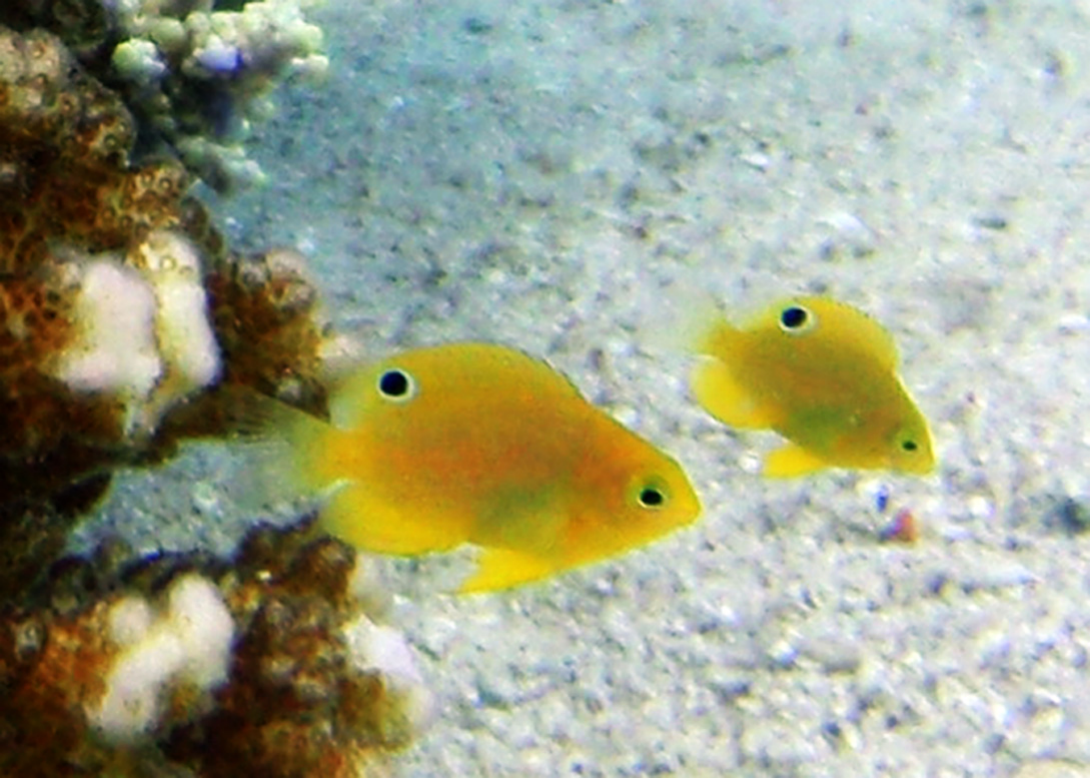
Newly settled juvenile ambon damselfish, *Pomacentrus amboinensis*. These are a common member of species diverse coral reef fish communities.

The ambon damselfish, *Pomacentrus amboinensis* is a site-faithful damselfish that is common on the shallow reefs of the Indo-Pacific. Adults and juveniles occur in shallow lagoons, where they inhabit the reef edge or reef top associated with rubble. Larval duration is 15–23 d with fish reaching 10–15 mm standard length (SL) at the end of the larval stage (Kerrigan 1996). Juvenile fish (Fig. 1) settle as solitary individuals into habitats with conspecific adults and subadults. These habitat patches can vary markedly in topographic complexity (McCormick and Weaver 2012).

Newly settled *P. amboinensis* are subject to an array of resident and transient predators. The dottyback, *Pseudochromis fuscus*, was used as the stimulus predator. It is a voracious and common predator of juvenile reef fishes and lives in the same habitat as the damselfishes (Feeney et al. 2012). These were collected from the reef with the aid of a dilute clove oil anesthetic and a hand net and maintained individually in 35 L flow-through aquaria with shelter, where they were fed one damselfish recruit (not necessarily *P. amboinensis*) twice per day prior to their use in experiments.

During November and December 2011, light traps (see Fig. 1 in Meekan et al. 2001 for design) were used to collect juvenile *P. amboinensis* at the end of their larval phase. Traps were moored at least 100 m away from the reef edge overnight, and catches were brought back to the Lizard Island Research Station just after dawn. Fish were placed into 60 L aquaria with aerated flowing seawater for 24 h (density ∼ 50 to 100 per 60 L) where they were fed *Artemia* twice per day for one to 2 weeks after which they were used in the laboratory experiments. The newly settled fish used in the study were of standard length (SL; 12.04 ± 0.05 mm; mean ± SE), and there was no difference in size among experimental treatments.

Studies of coral reef fishes have found that the pairing of skin extract from prey with a novel predator odor results in an antipredator response in conspecific prey upon exposure to the novel odor alone (Larson and McCormick 2005; Holmes and McCormick 2010). Fish can also learn the visual identity of a novel predator by the pairing of a conspecific skin extract with the sight of the predator (Chivers and Smith 1994; Ferrari et al. 2010a). After one to 2 weeks within the holding tanks, *P. amboinensis* were conditioned to associate the sight of *P. fuscus* as a threat by placing the predator in a bag within the tank, together with the damaged skin extract of two *P. amboinensis* (prepared as per protocol below). This standardized the *P. amboinensis* juveniles for predatory experience and ensured that all potential prey fish recognized *P. fuscus* as a risk to reduce experimental variance.

### Experimental protocol

One day after predator conditioning, individual *P. amboinensis* were placed into 15 L aquaria (38 × 27x24 cm; one fish per tank) with a constant flow of seawater and allowed to acclimate overnight. The basic tank setup included a 2 cm depth of coral sand and a small piece of dead *Pocillopora damicornis* coral skeleton (20 × 4 × 8 cm) placed against one end of the tank for shelter, while a single air tube was placed at the other end. A second tube was fixed to the aeration tube and allowed the introduction of *Artemia* food or chemical cues. The air facilitated the distribution of the cues throughout the tank (dye trials showed that it took 31.4 ± 0.9 s, and there was no difference between treatments). Each tank was surrounded on four sides with black plastic to prevent distractions to the focal fish. Treatment tanks were placed in alternative sequence on a bench exposed to natural lighting.

**Figure 2:**
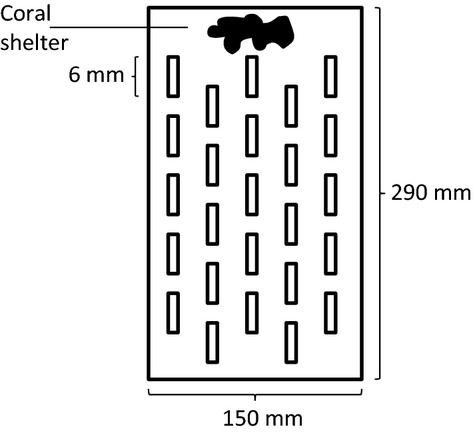
The experimental setup of the two different topography treatments in the laboratory. Structure was manipulated through the addition of a grid of baffles (190 mm × 20 mm by 6 mm) glued to a base (290 mm × 150 mm) as 5 rows of 5 baffles. These were either Perspex (i.e., transparent) or solid gray PVC.

There were two components of topography that were accounted for in this experiment: structure and visual obstruction. Three topography treatments were produced through the addition of structural complexity to this basic tank design: 1) no topography, consisting of the basic tank design as described above; 2) high structure but no visual barrier; 3) high structure and visual barrier. Structure was manipulated through the addition of a grid of baffles (190 mm × 20 mm by 6 mm) glued to a base (290 mm × 150 mm) as 5 rows of 5 baffles (Fig. 2). High structure but no visual barrier was achieved by making the baffles from clear Perspex, while in the high visual barrier treatment, baffles were constructed of gray PVC. Baffles were oriented long side to the long axis of the 15 L tank and arranged such that fishes would not be visible if they were three or more baffles into the tank (Figure S2). The base was buried in sand. A mirror was suspended over each tank at 45˚ so that focal fish could be observed undisturbed from above. A wire grid (3 × 3 cm grid size) was also placed on the top of the tank so that movement and location of individuals could be accurately quantified as the number of times fish crossed a line on the grid.

Prior to the start of the trial, the water flow was stopped and 5 mL of *Artemia* sp (∼550) nauplii was added to the aquaria to stimulate feeding. The behavior of a single *P. amboinensis* was recorded for a 4-min prestimulus period (only one fish per tank). Immediately following the prestimulus period, a further 5 mL of *Artemia* was added and fish were then exposed to one of 5 different cue treatments. The five chemical cues or visual stimuli added to each of the three tank designs were as follows: 1) damage-released conspecifics cues; 2) seawater (controlling for the addition of a fluid); 3) visual presence of a predator (*P. fuscus*) in a plastic bag; 4) visual presence of an empty plastic bag (control for disturbance of placing the predator next to the tank); and 5) combined effect of the presence of a damage-released cue and the sight of a predator. The behavior of the fish was then recorded for a further 4 min. Chemical alarm cues were prepared by euthanizing a *Pomacentrus amboinensis* juvenile by cold shock and superficially lacerating the flank 6 times. This lacerated area was rinsed with 10 mL of saltwater collected from the experimental aquaria and filtered prior to being used in the experiment. This solution of damage-released cues and seawater was then introduced into the tank through the second tube attached to the air tube with a syringe (Lönnstedt and McCormick 2011). Predators were placed in a clip-seal bag with a small amount of seawater to reduce the space available for movement. The bag containing the predator (or empty bag control) was carefully slipped between the outer side of the tank and black plastic tank blind (parallel to the longest surface of the baffles) to initiate the visual stimulus.

The behavioral response to experimental treatments was quantified by recording the following: total number of feeding strikes (successful or otherwise), activity (quantified as the number of times a fish crossed a line on the grid (3 × 3 cm) suspended over the tank), and time spent within shelter (defined as being inside the branches of the coral shelter). Fifteen replicates were run for each of the 15 treatment combinations (3 tank structures × 5 cues), and fish were not reused.

### Statistical analyses

A one-factor MANOVA was used to test whether the behavior of fish differed among the three levels of topographic complexity (empty, clear baffles, and solid baffles) prior to the addition of experimental stimuli. The dependent variables included in the analysis were bite rate and line crosses. Post hoc ANOVAs were undertaken to determine the nature of the differences in individual dependent variables found by MANOVA.

A two-factor MANOVA tested whether the behavior of fish differed among the three levels of topographic complexity and 5 stimuli (damage-released chemical cues, saltwater, sight of a predator, sight of an empty bag, and damage-released cues with the sight of a predator) or whether behavior was affected by the interaction between the two factors. The dependent variables included in the analysis were bite rate and line crosses. Change in behavior between pre- and poststimulus observations was used as the raw data. Significant effects in ANOVAs were further explored using unequal-sample Tukey's HSD tests. Assumptions of homogeneity of variance and normality were examined with residual analysis. The change in time spent in shelter intractably violated analysis assumptions due to the high number of zero values in the controls (because fish did not change their shelter use in response to seawater); hence, it was not included in the MANOVA. Seawater and empty bag treatments were dropped for this variable, and a two-factor ANOVA was then run on the remaining cue and topographic complexity treatments.

## Results

Topographic complexity affected the behavior of fish prior to the addition of cues (MANOVA, Pillai's Trace = 0.072, df 6,442, *P* = 0.012). Univariate exploration showed that the effect of complexity was driven by fish within the low complexity tanks having higher feeding rates than those in the other two treatments (mean bite rate per 4 min: 69, no complexity; 63, clear baffles; 64, gray baffles). Line crosses were not significantly affected by the three different complexity treatments (*P* > 0.05).

**Figure 3:**
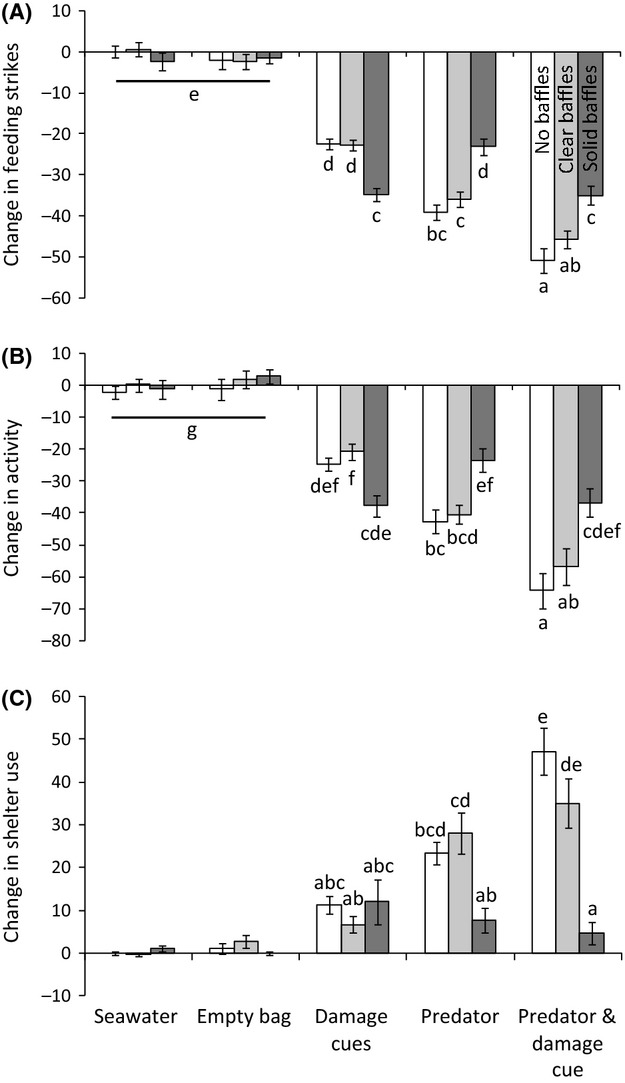
Effects of three levels of topographic habitat complexity on risk assessment by a juvenile damselfish, *Pomacentrus amboinensis*. Graphs show the change (±SE) in behavior in response to two control treatments (addition of seawater and visual presentation of an empty plastic bag) and three cue treatments (chemical cues from damaged skin; visual presentation of a predator in a plastic bag; and combination of the two). Each cue is given in one of three tank environments (tank with no baffles; tank with clear baffles; and tank with solid baffles that restricted vision). Variables presented are as follows: (A) change in feeding strikes (bites per 4 min); (B) activity (line crosses per 4 min); (C) shelter use (% time among coral branches). Change represents the difference in magnitude of a variable between 4 min observations before the introduction of a cue and after introduction, so negative values represent a reduction in the variable in response to the cue. Letters above or below bars indicate Tukey's HSD post hoc groupings (*n* = 15).

Topographic complexity and the cue interacted together to affect the behavior of the juvenile fish (MANOVA, Pillai's Trace = 0.355, df 16,420, *P* < 0.0001). ANOVAs found that the multivariate interaction was driven by interactions in both variables measured in the analysis. Feeding strikes and line crosses showed exactly the same pattern of response (Fig. 3a,b; Interaction feeding strikes *F*_8,210_ = 11.614, *P* < 0.0001; line crosses *F*_8,210_ = 6.741, *P* < 0.0001). The control treatments (seawater and empty bag) did not affect fish behavior regardless of the complexity within the tank (Fig. 3a,b). Damaged-released cues and the sight of a predator reduced feeding and line crosses and appeared to have an additive effect in concert when fish had no structure in their tank, or baffles were transparent (Fig. 3a,b).

When fish were in a tank with the gray PVC baffles (high complexity), they displayed a significant reduction in feeding strikes and line crosses in response to the damage-released cues compared with when no visual barriers were present (Tukey's test *P* < 0.001, Fig. 3a,b). When exposed to the sight of a predator, fish in the complex habitat also displayed a lower reduction in feeding strikes and activity compared with controls (Tukey's tests *P* < 0.03; Fig. 3a,b). Furthermore, when exposed to a combination of the sight of a predator and a damage-released cue fish in the high complexity, treatment displayed a reduction in feeding and activity that was the same magnitude as the response to damage-released cues on their own (Fig. 3a,b).

There was a significant interaction between the tank topographic complexity and cue on the time fish spent in the shelter of the coral branches (*F*_4,126_ = 9.176, *P* < 0.0001). The injection of seawater and the sight of an empty plastic bag did not alter the percentage of time fish spent in the shelter (Fig. 3c). Fish displayed a similarly small increase in shelter use when exposed to damage-released cues regardless of topographic complexity (Fig. 3c). The sight of a predator resulted in a ∼25% increase in shelter use in the treatments where the sight of the predator was not obscured by structure. Although fish from these low complexity treatments displayed a more than 30% increase in shelter use when exposed to a combination of damaged-released cues and the sight of a predator, the response did not differ from when they could only see the predator (Fig. 3c, Tukey's results). Fish in the high complexity treatment did not significantly increase their shelter usage in response to either chemical, visual, or the combination of risk indicators above that of the controls (Fig. 3c).

## Discussion

The present study indicates that juvenile fish are overall more cautious in high topography environments, with prey having lower feeding rates and relying on olfactory cues for risk assessment. Importantly, topographic complexity affected the way the prey fish assessed risk and responded to predation threats. Fish appeared to compensate for a reduction in the visual information available to them in a high topography environment by relying more on olfactory information. Previous studies have argued that chemical information should be more important than visual information in the assessment of risk where vision is obscured by topographic complexity (e.g., McCormick and Manassa 2008; Lönnstedt et al. 2012), turbidity (Ferrari et al. 2010b; Leahy et al. 2011) or low or no light (Chivers et al. 1996; Leduc et al. 2010), and our evidence supports these assertions.

Topographically complex habitats limit visual cues and aid cryptic ambush predators (Schultz and Kruschel 2010), and prey would be expected to instinctively display more conservative behaviors within these habitats; foraging rates were lower in the high complexity tanks prior to the addition of cues, and they displayed a greater response to an olfactory indicator of threat. Our experiment found that prey alter the balance between foraging and other fitness-related activities in favor of higher vigilance when topographic habitat complexity is high. It is expected that prey will best be able to assess types of predators and the nature of the risk posed when information from multiple sensory modes is available (Amo et al. 2006). Chemical cues may remain well after a predator has left the vicinity, and a reliance on chemical information alone may lead to an overestimation of risk (Turner and Montgomery 2003). Visual identification of the predator and its attributes (e.g., size, behavior) provide information on the predator's level of motivation and threat (e.g., Helfman 1989; Smith and Belk 2001), and so vision is usually the most reliable sense in the detection of predators (Cronin 2005). Lima and Steury (2005) proposed the “sensory complement” hypothesis whereby prey would respond to multiple cues containing information about risk in an additive manner. With vision obscured in the topographically complex habitats, prey had less information on which to base their assessment of predation risk and were less responsive to threats than when visual information was available. Given this, we predict a dome-shaped relationship between foraging (and subsequent growth) and topographic complexity on the scale of the home range for diurnally active organisms, with optimum amount of shelter associated with the highest foraging rates; enough topography to provide shelter but not so much that it obscures vision. Given only two levels of structure and visual barrier were used in the present study, further studies are required using more levels of topography to test this prediction.

The provision of damage-released cues and sight of a predator resulted in a nonadditive response in the high topography environment as the visual cues were impeded by the complexity of the habitat. Thus, there was a stronger reliance on olfactory information in topographically complex habitats (such as healthy coral reef environments). Such sensory compensation has been previously shown in circumstances when the balance of sensory cues is altered due to changes in the transparency of the environment to particular cue types. Hartman and Abrahams (2000) found sensory compensation occurred between visual and olfactory senses in fathead minnows (*Pimephales promelas*). Minnows in turbid water relied more on chemical information because their vision was impaired. Similar results were recently found for a tropical damselfish (Leahy et al. 2011). Chivers et al. (2001) noted that in clear water, chemical signals were less important if not associated with visual cues of predator activity. Leduc et al. (2010) found that juvenile Atlantic salmon responded with a higher intensity to chemical indicators of threat at night than during the day when fish were able to visually assess threat. Obviously, there are many instances where fishes will shift the balance of cues used to assess risk to maximize the amount of unique information on which to base a behavioral decision. Topography appears to be a variable that will be important in altering the balance of cues used in bottom dwelling fishes and is likely to be most relevant to diurnally active fishes. The degree of flexibility that individuals and species have in their ability to adjust the balance of senses used in risk assessment will determine the extent to which organisms will tolerate modifications to their environment through habitat disturbance.

Our experiments suggest that topographic complexity influenced risk assessment through its impact on sensory cues rather than through the physical structure restricting movement. Restrictions to navigation may be expected to make the prey more conservative because structure should hamper their escape path (Drolet et al. 2004); however, there was no evidence of this effect. In fact, the higher complexity of the habitat (containing numerous crevices and shelter spots) may allow prey to feel safer and more protected against bottom dwelling predators. This may be an alternative explanation for the limited reduction in activity in high complexity treatment in response to the sight of a predator in the present study although it does not explain the heightened response to olfactory indicators of threat. Indeed, familiarity with the structural layout within a home range has been shown to be beneficial for avoiding and escaping predators (Aronson 1971; Strauss et al. 2008). Thus, while physical structures did not appear to play an important role in risk assessment in the present experiment, it may play a key role through influencing outcome of an encounter of a predator with prey in a complex environment once direct interaction is initiated (Drolet et al. 2004).

Topographic complexity is expected to affect the movement of currents in the vicinity of structures, which are the areas inhabited by juvenile fishes, who are particularly vulnerable to predation (Almany and Webster 2006). Barriers and surface rugosity affect the passage of water over surfaces and influence small-scale hydrological features such as the thickness of the boundary layer, turbulence, and speed of the water flow (Weissburg 2000). The magnitude of the effects of topography on flow is dependent upon overall current speed (Abelson and Denny 1997). Anything that affects current at the scale that organisms receive olfactory cues is likely to affect the use of this mode for risk assessment and its utility in relation to other sensory modes.

The current experiment manipulated topography using artificial structures to isolate the effects of topographic structures from a visual barrier. However, in the natural environment, as hard coral degrades from live to dead coral through to rubble, topographic complexity is not the only parameter that changes that will influence the balance of cues used for risk assessment. A recent study has found that the odors released from dead, algae covered coral habitats alter the prey damage-released cues that are normally reliable indicators of predation risk (Lönnstedt et al. 2013). This alteration of the cue effectively prevents fish from detecting damaged conspecifics using olfactory means and eliminates the important role that these cues play in learning the identity of novel predators (Ferrari et al. 2010a) and the dynamic adjustment of the risk through cue reinforcement and latent inhibition (Mitchell et al. 2011). Thus, while the degradation of hard corals will reduce habitat topography and increase the breadth of sensory cues available to risk assessment by increasing the availability of visual information, in coral reef environments, it will come at a cost of the reduction in the efficacy of olfactory cues. This may reduce survival in degraded habitat, contrary to the expectations of moderate decreases in topographic complexity (Lönnstedt et al. 2013).

Our study has illustrated the importance of the visual barrier represented by topographic complexity to the balance of senses used in risk assessment. Whether the impaired visual mode in high topography elevates mortality rates is unknown without consideration of how predator foraging efficiency may be affected by topography. This will be determined by not only how the prey use topography to evade detection and escape attack once detected, but how the predator uses the visual and structural properties of topography to stalk and capture prey. In today's changing world, we know little of how habitat degradation will affect predator–prey interactions. If prey display a stronger reliance on visual cues in low complexity dead habitats on coral reefs, they may be able to escape an otherwise certain death in habitats where olfactory information is diminished.

## Supporting Information

**Figure S1–S2.** Topographic complexities in the field and laboratory.

**Figure S1.** The difference between (A) live, healthy coral reef environment (topographically complex; containing numerous crevices and holes to hide in) and that of a (B) degraded, low complexity reef.

**Figure S2.** The three different topography treatments: 1) no topography, consisting of the basic tank design as described in the main text; 2) high structure but no visual barrier; 3) high structure and a visual barrier.

## References

[b1] Abelson A, Denny M (1997). Settlement of marine organisms in flow. Ann. Rev. Ecol. System.

[b2] Almany GR, Webster MS (2006). The predation gauntlet: early post-settlement mortality in coral reef fishes. Coral Reefs.

[b3] Alvarez-Filip L, Dulvy NK, Gill JA, Côté IM, Watkinson AR (2009). Flattening of Caribbean coral reefs: region-wide declines in architectural complexity. Proc. Roy. Soc. Lond. B.

[b4] Amo L, López P, Martín J (2006). Can wall lizards combine chemical and visual cues to discriminate predatory from non-predatory snakes inside refuges?. Ethology.

[b5] Anthony KRN, Maynard JA, Diaz-Puildo G, Mumby PJ, Marshall PA, Cao L (2011). Ocean acidification and warming will lower coral reef resilience. Global Change Biol.

[b6] Aronson LR (1971). Further studies on orientation and jumping behaviour in the gobiid fish, *Bathygobius soporator*. Ann. New York Acad. Sci.

[b7] Brodie J, Post E, Laurance WF (2012). Climate change and tropical biodiversity: a new focus. Trends Ecol. Evol.

[b8] Chivers DP, Smith RJF (1994). Fathead minnows, *Pimephales promelas*, acquire predator recognition when alarm substance is associated with the sight of unfamiliar fish. Anim. Behav.

[b9] Chivers DP, Brown GE, Smith RJF (1996). The evolution of chemical alarm signals: attracting predators benefits alarm signal senders. Am. Nat.

[b10] Chivers DP, Mirza RS, Bryer PJ, Kiesecker JM (2001). Threat-sensitive predator avoidance by slimy sculpins: understanding the importance of visual versus chemical information. Can. J. Zool.

[b11] Clinchy M, Sheriff MJ, Zanette LY (2013). Predator-induced stress and the ecology of fear. Funct. Ecol.

[b12] Creel S, Christianson D (2007). Relationships between direct predation and risk effects. Trends Ecol. Evol.

[b13] Cronin TW, Barbosa P, Castellanos I (2005). The visual ecology of predator-prey interactions. Ecology of Predator/Prey Interactions.

[b14] Drolet D, Himmelman JH, Rochette R (2004). Use of refuges by the ophiuroid *Ophiopholis aculeate*: contrasting effects of substratum complexity on predation risk from two predators. Mar. Ecol. Prog. Ser.

[b15] Feeney WE, Lönnstedt OM, Bosiger YJ, Martin J, Jones GP, Rowe RJ (2012). High rate of prey consumption in a small predatory fish on coral reefs. Coral Reefs.

[b16] Ferrari MCO, Vavrek M, Elvidge C, Fridman B, Chivers DP, Brown GE (2008). Sensory complementation and the acquisition of predator recognition by salmonid fishes. Behav. Ecol. Sociobiol.

[b17] Ferrari MCO, Wisenden BD, Chivers DP (2010a). Chemical ecology of predator-prey interactions in aquatic ecosystems: a review and prospectus. Can. J. Zool.

[b18] Ferrari MCO, Lysak KR, Chivers DP (2010b). Turbidity as an ecological constraint on learned predator recognition and generalization in a prey fish. Anim. Behav.

[b19] Hartman EJ, Abrahams MV (2000). Sensory compensation and the detection of predators: the interaction between chemical and visual information. Proc. Roy. Soc. Lond. B.

[b20] Helfman GS (1989). Threat-sensitive predator avoidance in damselfish-trumpetfish interactions. Behav. Ecol. Sociobiol.

[b21] Hewitt JE, Thrush SE, Halliday J, Duffy C (2005). The importance of small-scale habitat structure for maintaining beta diversity. Ecology.

[b22] Holmes TH, McCormick MI (2010). Smell, learn and live: the role of chemical alarm cues in predator learning during early life history in a marine fish. Behav. Processes.

[b23] Hughes TP, Graham NAJ, Jackson JBC, Mumby PJ, Steneck RS (2010). Rising to the challenge of sustaining coral reef resilience. Trends Ecol. Evol.

[b24] Jones GP, McCormick MI, Srinivasan M, Eagle JV (2004). Coral decline threatens fish biodiversity in marine reserves. Proc. Nat. Acad. Sci.

[b25] Kerrigan BA (1996). Temporal patterns in the size and condition of settlement in two tropical reef fishes (Pomacentridae: *Pomacentrus amboinensis* and *P. nagasakiensis*. Mar. Ecol. Prog. Ser.

[b26] Larson JK, McCormick MI (2005). The role of chemical alarm signals in facilitating learned recognition of novel chemical cues in a coral reef fish. Anim. Behav.

[b27] Laundré JW, Hernández L, Altendorf KB (2001). Wolves, elk, and bison: reestablishing the “landscape of fear” in Yellowstone National Park, U.S.A. J. Zool.

[b28] Leahy SM, McCormick MI, Mitchell M, Ferrari MCO (2011). To fear or to feed: the effects of turbidity on perception of risk by a marine fish. Biol. Let.

[b29] Leal IR, Filgueiras BKC, Gomes JP, Iannuzzi L, Andersen AN (2012). Effects of habitat fragmentation on ant richness and functional composition in Brazilian Atlantic forest. Biodiv. Conserv.

[b30] Leduc AOHC, Kim J-W, MacNaughton CJ, Brown GE (2010). Sensory complement model helps to predict diel alarm response patterns in juvenile Atlantic salmon (*Salmo salar*) under natural conditions. Can. J. Zool.

[b31] Lima SL, Steury TD, Barbosa P, Castellanos I (2005). The perception of predator risk - the foundation of non-lethal predator-prey interactions. Ecology of Predator/Prey Interactions.

[b32] Lönnstedt OM, McCormick MI (2011). Chemical alarm cues inform prey of predation threat: the importance of ontogeny and concentration in a coral reef fish. Anim. Behav.

[b33] Lönnstedt OM, McCormick MI, Meekan MG, Ferrari MCO, Chivers DP (2012). Learn and live: the role of predator experience in influencing prey behaviour and survival. Proc. Roy. Soc. Lond. B.

[b34] Lönnstedt OM, McCormick MI, Chivers DP (2013). Habitat degradation affect prey risk assessment. Ecol. Evol.

[b35] McCormick MI, Manassa R (2008). Predation risk assessment by olfactory and visual cues in a coral reef fish. Coral Reefs.

[b36] McCormick MI, Weaver C (2012). It pays to be pushy: intracohort interference competition between two reef fishes. PLoS ONE.

[b37] Meekan MG, Wilson SG, Halford A, Retzel A (2001). A comparison of catches of fishes and invertebrates by two light trap designs, in tropical NW Australia. Mar. Biol.

[b38] Metcalfe NB (1984). The effects of habitat sensory compensation and the detection of predators: the interaction between chemical and visual information. Proc. Roy. Soc. B.

[b39] Mitchell MD, McCormick MI, Ferrari MCO, Chivers DP (2011). Friend or foe? The role of latent inhibition in predator and non-predator labelling by coral reef fishes. Anim. Cogn.

[b40] Moran PJ (1986). The *Acanthaster* phenomenon. Oceanogr. Mar. Biol. Ann. Rev.

[b41] Mumby PJ, Vitolo R, Stephenson DB (2011). Temporal clustering of tropical cyclones and its ecosystem impacts. Proc. Nat. Acad. Sci.

[b42] Munday PL, Jones GP, Pratchett MS, Williams AJ (2008). Climate change and the future for coral reef fishes. Fish Fish.

[b43] Pratchett MS, Munday PL, Wilson SK, Graham NAJ, Cinner JE, Bellwood DR (2008). Effects of climate-induced coral bleaching on coral-reef fishes - ecological and economic consequences. Oceanogr. Mar. Biol.

[b44] Pratchett MS, Wilson SK, Graham NAJ, Munday PL, Jones GP, Polunin NVC, van Oppen MJH, Lough JM (2009). Coral bleaching and consequences for motile reef organisms: past, present and uncertain future effects. Coral Bleaching: Patterns, Processes, Causes and Consequences.

[b45] Schoener TW, Spiller DA, Losos JB (2002). Predation on a common Anolis lizard: can the food-web effects of a devastating predator be reversed?. Ecol. Monogr.

[b46] Schultz ST, Kruschel C (2010). Frequency and success of ambush and chase predation in fish assemblages associated with seagrass and bare sediment in an Adriatic lagoon. Hydrobiologia.

[b47] Smith ME, Belk MC (2001). Risk assessment in western mosquitofish (*Gambusia affinis*): do multiple cues have additive effects?. Behav. Ecol. Sociobiol.

[b48] Strauss A, Solmsdorff KY, Pech R, Jacob J (2008). Rats on the run: removal of alien terrestrial predators affects bush rat behaviour. Behav. Ecol. Sociobiol.

[b49] Thompson AA, Dolman AM (2010). Coral bleaching: one disturbance too many for near-shore reefs of the Great Barrier Reef. Coral Reefs.

[b50] Turner AM, Montgomery SL (2003). Spatial and temporal scales of predator avoidance: experiments with fish and snails. Ecology.

[b51] Weissburg MJ (2000). The fluid dynamical context of chemosensory behavior. Biol. Bull.

[b52] Willis BL, Page CA, Dinsdale EA, Rosenberg E, Loya Y (2004). Coral disease on the Great Barrier Reef. Coral Health and Disease.

[b53] Wilson SK, Graham NAJ, Pratchett MS, Jones GP, Polunin NV (2006). Multiple disturbances and the global degradation of coral reefs: are reef fishes at risk or resilient?. Global Change Biol.

[b54] Wilson SK, Dolman AM, Cheal AJ, Emslie MJ, Pratchett MS, Sweatman HPA (2009). Maintenance of fish diversity on disturbed coral reefs. Coral Reefs.

