# Supplementary material for: Degrading habitats and the effect of topographic complexity on risk assessment
Source: Ecol Evol. 2013 Sep 30;3(12):4221–9. doi: 10.1002/ece3.793 (PMC3853566; doi:10.1002/ece3.793)
Supplement: Supplementary file 1 [file ece30003-4221-SD1.pdf]

Supplementary information

McCormick, M. I., Lönnstedt, O. M. Degrading habitats and the effect of topographic complexity on risk assessment. Ecology and Evolution.

Supplementary 1. Topographic complexities in the field and laboratory

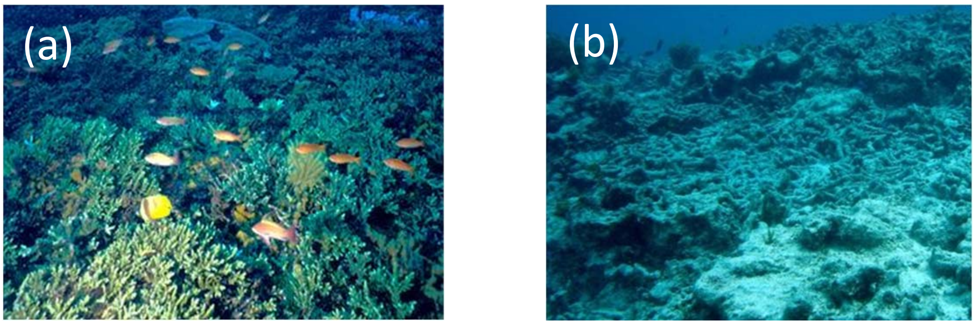

Figure S1. The difference between (a) live, healthy coral reef environment (topographically complex; containing numerous crevices and holes to hide in) and that of a (b) degraded, low, complexity reef

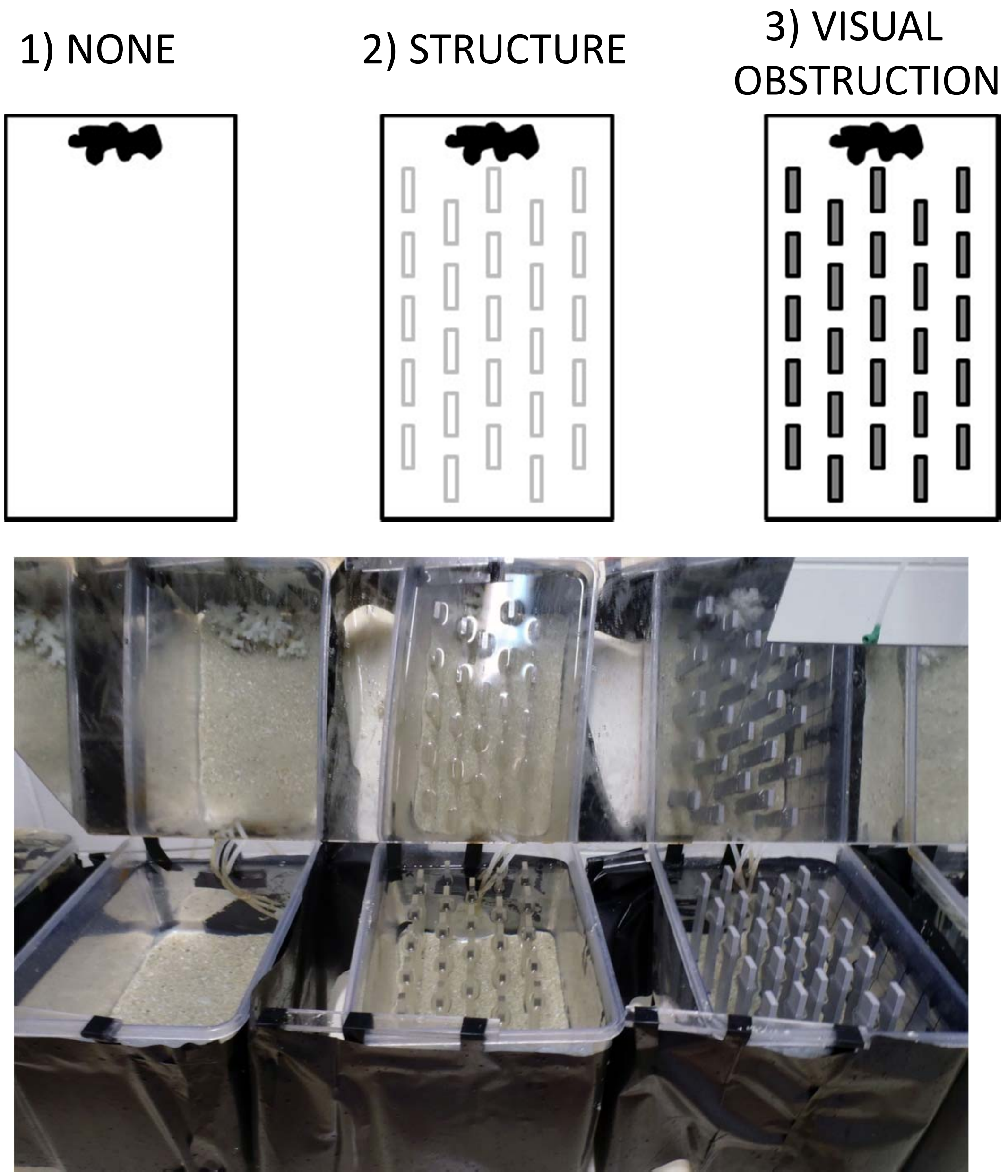

Figure S2. The three different topography treatments: 1) No topography, consisting of the basic tank design as described above; 2) High structure but no visual barrier; 3) High structure and a visual barrier. High structure but no visual barrier was achieved by making the baffles from clear Perspex, while in the high visual barrier treatment baffles were constructed of grey PVC. A mirror was suspended over teach tank at 45˚ so that focal fish could be observed undisturbed from above.
